# The establishment of a rheumatoid arthritis primate model in *Macaca fascicularis*

**DOI:** 10.1186/s12967-020-02402-z

**Published:** 2020-06-30

**Authors:** Hyun Sik Na, Seon-yeong Lee, Hong Ki Min, Wan-je Park, Jung-hwan Lee, Ka-hee Cho, Shin-hee Hong, Dae-hoon Kim, Jooyeon Jhun, Jeong-Won Choi, Sung-Min Kim, Seung-Ki Kwok, Mi-La Cho, Sung-Hwan Park

**Affiliations:** 1 Rheumatism Research Center, Catholic Research Institute of Medical Science, College of Medicine, The Catholic University of Korea, Seoul, 06591 Korea; 2 Laboratory of Immune Network, Catholic Research Institute of Medical Science, College of Medicine, The Catholic University of Korea, Seoul, Republic of Korea; 3 Department of Medical Lifescience, College of Medicine, The Catholic University of Korea, 222, Banpo-daero, Seocho-gu, Seoul, 06591 Republic of Korea; 4 Division of Rheumatology, Department of Internal Medicine, Konkuk University Medical Center, Seoul, Republic of Korea; 5 Division of Rheumatology, Department of Internal Medicine, Seoul St. Mary’s Hospital, College of Medicine, The Catholic University of Korea, Seoul, South Korea; 6 Haeeun Biomedical Research Institute, Genia Inc, Sungnam, Korea; 7 Conversant Research Consortium in Immunologic Disease, College of Medicine, The Catholic University of Korea, 505 Banpo-Dong, Seocho-Ku, 137-040 Seoul, Korea

**Keywords:** Rheumatoid arthritis, Primate model, *Macaca fascicularis*, Type II collagen

## Abstract

**Background:**

Rheumatoid arthritis (RA) is a long-term autoimmune disorder that mostly affects the joints and leads to the destruction of cartilage. An RA model in non-human primates is especially useful because of their close phylogenetic relationship to humans in terms of cross-reactivity to compounds developed using modern drug technologies.

**Methods:**

We used a collagen-induced arthritis (CIA) model in *Macaca fascicularis*. CIA was induced by the immunization of chicken type II collagen. Swelling was measured as the longitudinal and transverse axes of 16 proximal interphalangeal joints.

**Results:**

A new system for visual evaluation was created, with a perfect score of 16. Individual behavioral analysis was also conducted. Serum was collected once a week after the first immunization. Blood chemistry and inflammatory cytokine parameters were higher in the CIA group than in the wild type group.

**Conclusion:**

In conclusion, we established CIA in *M. fascicularis*, and the results can be used for drug evaluation models.

## Background

Rheumatoid arthritis (RA) is an autoimmune-mediated inflammatory disease. Some pathogenic mechanisms of RA have been revealed, and several novel treatment modalities, based on blocking pro-inflammatory cytokines or modulating intra-cellular signals such as Janus kinase (JAK)/signal transducer and activator of transcription proteins (STATs), are used nowadays [1]. Despite advances in our understanding of RA pathogenesis and improvements in RA treatment, some RA patients are refractory to conventional disease-modifying anti-rheumatic drugs (DMARDs), biological DMARDs, and even to the most recently developed small-molecule treatments, such as JAK inhibitors. To find solutions for these refractory RA patients, research into novel pathogenic mechanisms of RA and treatment strategies for RA are actively on-going.

To develop and prove the efficacy of new medication for RA patients, several stepwise pre-clinical and clinical studies are required. Most of the pre-clinical studies are performed on rodent models of RA, such as collagen-induced arthritis (CIA) or antibody-induced arthritis. Rodents are relatively easy to manage and are more economical than primates; however, rodent models have a critical failing due to the differences between humans and rodents in terms of morphology, physiology, and phylogenetic closeness [2, 3]. Therefore, any beneficial effects proven in rodent models do not guarantee the same or similar therapeutic effects in RA patients. There is an unmet need to establish a better animal model for RA to enhance our knowledge of RA pathogenesis and to conduct pre-clinical experiments using an RA animal model that is similar to humans.

Non-human primates are an ideal animal model to investigate RA pathogenesis and treatment responses because they resemble humans more closely than rodents in terms of their physiology, morphology, and genetic background. Several non-human primate background RA models have been introduced, however, there are some limitations. The rhesus monkey, *Macaca mulatta*, was first used as an animal RA model; however, some individuals had specific major histocompatibility complex (MHC) class I alleles that were resistant to the induction of arthritis via type II collagen injections [4, 5]. These CIA models, based on *M. mulatta*, had only a 70% induction rate for arthritis [4]. The cynomolgus monkey, *M. fascicularis*, has emerged as an alternative primate animal model for RA [6–9], however, a standardized scoring system has not yet been established for this model. Furthermore, osteoporosis is one of the major co-morbidities of RA [10], and an evaluation of the degree of osteoporosis, such as bone density in primates models of RA, has not been conducted previously.

In this study, we induced CIA in *M. fascicularis* and established a scoring system of various clinical markers. Furthermore, we also investigated the MHC class I allele responsible for arthritis resistance in the *M. mulatta*. Finally, we performed micro-computed tomography (micro-CT) of the paw to evaluate the induction of osteoporosis in a primate model of RA.

## Methods

### Animals

*Macaca fascicularis* monkeys weighing 3–4 kg (age, 3–5 years) at the start of the experiment were purchased from GENIA (Seongnam, South Korea). Animals were housed in single cages in a controlled temperature room (21–29 °C) and light (12-h light–dark cycle) conditions. The monkeys had free access to a gamma-ray-sterilized diet (5048, LabDiet, St. Louis, MO, USA) and autoclaved R/O water. All animal research procedures were conducted in accordance with the Laboratory Animals Welfare Act, the Guide for the Care and Use of Laboratory Animals, and the Guidelines and Policies for Non-human Primate (NHP) Experiments provided by the Institutional Animal Care and Use Committee (ORIENT-IACUC-16255).

### fascicularis B*01 gene analysis

Total mRNA was isolated from B cells of each *M. fascicularis* monkey and converted to cDNA. The cDNA was subjected to polymerase chain reaction (PCR) to identify the CIA-related B*01 gene. The following B-specific primers were used: MBS (sense) 5′- AAT TCA TGG CGC CCC GAA CCC TCC TCC TGC -3′ and (anti-sense) 5′- CTA GAC CAC ACA AGA CAG TTG TCT CAG-3′.

### Induction of arthritis

CIA was induced in *M. fascicularis*. Type II collagen (CII) was dissolved overnight in 0.1 N acetic acid (4 mg/mL) with gentle rotation at 4 °C. Female *M. fascicularis* monkeys were immunized intradermally at the base of the tail with 100 µg of chicken CII (Chondrex Inc., Redmond, WA, USA) in complete Freund’s adjuvant (Chondrex Inc.). *M. fascicularis* were boosted with 400 µg of CII emulsified with incomplete Freund’s adjuvant (Chondrex Inc.) and injected intradermally into 10 sites on the back, 21 days after the primary immunization.

### Arthritis scoring

The arthritis score was created by grading for disease severity. We used four scoring methods. First, clinical scoring was conducted as follows: 0, no disease symptoms; 0.5, fever; 1, apathy, lessened mobility and loss of appetite; 2, weight loss, warm extremities, and treatable pain without sinus tarsi syndrome (STS); 3, redness of joints, normal flexibility of extremities; 4, severe STS of joints, joint stiffness; and 5, untreatable pain, immobility of joints, weight loss > 25%. Second, phenotype scoring was as follows: 0, no evidence of swelling; 1, over 0.5 cm swelling of the proximal interphalangeal (PIP) joint compared with week 0 (< 0.5 cm^2^); 2, score 1 evidence in two PIP joints; 3, score 1 evidence in three PIP joints; and 4, score 1 evidence in four PIP joints. Third, behavior scoring was as follows: 0, no evidence of behavior; 1, drag or limp in a hind leg; 2, slipping over in cage; and 3, one leg is not working. Fourth, pain scoring was as follows: 0, no evidence of pain; 1, tremor in paws; 2, tremor in paws and losing grip; and 3, severe tremor in paws and a crouching position.

### Histopathological analysis

The PIP joints were collected from each group at 8 weeks after the first immunization. The tissues were fixed in 10% neutral buffered formalin solution, decalcified using Decalcifying Solution-Lite (Sigma, St. Louis, MO, USA), and embedded in paraffin. Sections of 4- to 5-μm thickness were cut, dewaxed using xylene, dehydrated in an alcohol gradient, and stained with hematoxylin and eosin (H&E) and safranin O.

### Immunohistochemistry

Paraffin-embedded sections were incubated at 4 °C with the following primary monoclonal antibodies: anti-IL**-**1β, anti-IL**-**6 (Novus Biologicals, Littleton, CO, USA), anti-IL**-**17, and anti-TNF**-**α (Abcam, Cambridge, UK). The samples were then incubated with horseradish peroxidase-conjugated secondary antibody for 30 min. The reaction product was developed using 3,3-diaminobenzidine chromogen (Dako, Carpinteria, CA, USA). Histological assessments were conducted by three independent blinded observers. Images were captured using a DP71 digital camera (Olympus, Shinjuku, Tokyo, Japan) attached to a photomicroscope at a magnification of 200 × . Positively stained cells were counted using Adobe Photoshop software (Adobe, San Jose, CA, USA), and the mean values were calculated.

### Flow cytometry analysis

Peripheral blood mononuclear cells (PBMCs) were isolated at 8 weeks after induction of arthritis. To analyze the population of T helper cells, the isolated PBMCs were stained with anti-CD4 peridinin chlorophyll protein complex (PerCP; BD Biosciences, San Diego, CA, USA), anti-IFNγ-fluorescein isothiocyanate (FITC; BD Biosciences), and anti-IL-17A eFluor 660 (eBioscience, San Diego, CA, USA). For regulatory T cells, the PBMCs were stained with anti-CD4 PerCP (BD Biosciences), anti-CD25-allophycocyanin (APC; BD Biosciences), and anti-Foxp3-PE (BD Biosciences). Cells were permeabilized and fixed with CytoPerm/CytoFix (BD Biosciences) according to the manufacturer’s protocol. Flow cytometry was performed using a FACSCalibur cytometer (BD Biosciences).

### In vivo micro-CT imaging and analysis

Micro-CT imaging and analysis were performed using an animal scanner (SkyScan 1176, Billerica, MA, USA). The animals were imaged at settings of 60 kVp and 417 μA using an aluminum 0.5-mm thick filter. The pixel size was 35.76 μm, and the rotation step was 0.4°. Cross-sectional images were reconstructed using a filtered back-projection algorithm (NRecon Software, Bruker Micro CT, Kontich, Belgium). For each scan, a stack of 286 cross-sections was reconstructed at 4000 × 2670 pixels. Bone volume and bone surfaces were analyzed at the proximal interphalangeal and wrist joints.

### Enzyme-linked immunosorbent assay (ELISA)

The concentrations of IL-6 in the blood serum of *M. fascicularis* were measured using a sandwich ELISA (DuoSet; R&D Systems, Lille, France).

### Hematology and serum biochemistry

Hematology values were determined using a Cell-Dyn^®^ 3700 hematologic analyzer (Abbott Diagnostics, Wiesbaden, Germany), and serum biochemistry was evaluated using a Hitachi clinical analyzer 7180 (Hitachi Ltd., Tokyo, Japan). The hematology analysis included hemoglobin (Hb; g/dL), and biochemistry analysis included C-reactive protein (CRP; mg/dL).

### Statistical analysis

The results are expressed as mean ± standard deviation and were obtained from three separate experiments. Statistical significance was determined according to the Mann–Whitney U-test or an ANOVA with Bonferroni’s post hoc test, performed using GraphPad Prism (version 5.01, GraphPad Software, San Diego, CA, USA). A *p* value < 0.05 was considered to indicate statistical significance.

## Results

### Resistance to CIA by B*01 genotype

**Fig. 1:**
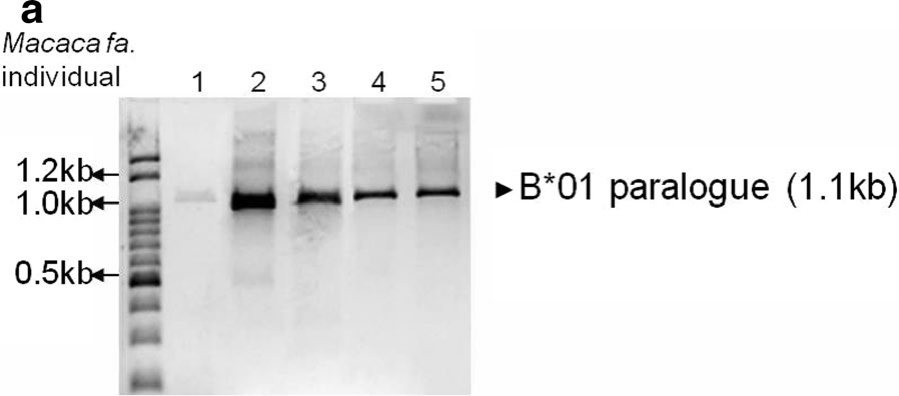
*Macaca fascicularis* B*01 genotyping results. The presence of the B*01 gene was analyzed by RT-PCR in *M. fascicularis.***a** Each cDNA was subjected to PCR to identify the CIA-related B*01 gene

*Macaca mulatta* monkeys are resistant to CIA when they have the B*01 genotype. However, we confirmed that *M. fascicularis* has no resistance to CIA [4, 11]. PCR products of 1.1 kb were detected, and sequenced data of each *M. fascicularis* included the *Gogo*-B*01 gene. The *M. fascicularis* had the B*01 gene among their MHC class I genes, but all were susceptible to CIA induction (Fig. 1).

### Clinical scoring of CIA in *M. fascicularis*

**Fig. 2:**
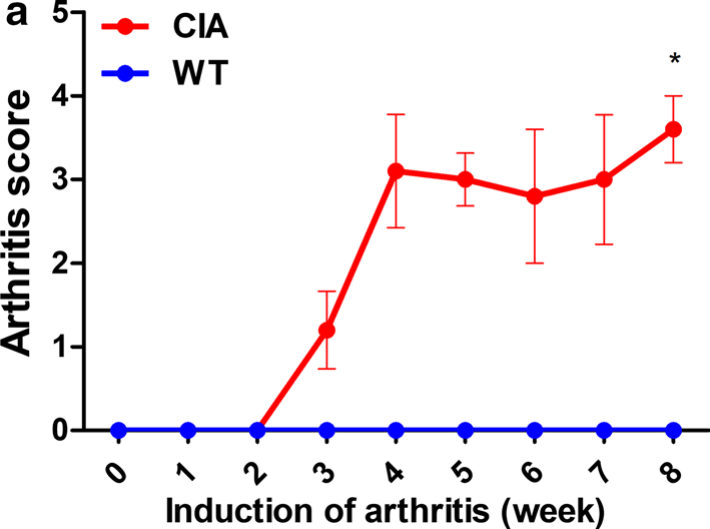
Clinical scoring of CIA in *M. fascicularis*. Each group was followed for 8 weeks. **a** The clinical score was assessed, as seen in Table 1 (***p < 0.001)

CIA monkeys were intradermally administered a CII/CFA emulsion. The second booster immunization was performed after 3 weeks. Clinical changes in the *M. fascicularis* CIA model were assessed with a clinical scoring system [12] (Table 1). Clinical symptoms of CIA were monitored weekly for 8 weeks after the first immunization. All clinical signs were monitored weekly and included body weight, body temperature, number of joints affected, soft tissue swelling, warmth, and redness. These CIA monkeys showed more severe CIA symptoms and a high clinical score compared to wild-type *M. fascicularis* (Fig. 2).

**Table 1:** Arthritis clinical scoreing

Disease score	Characteristics
0	No disease symptoms
0.5	Fever (> 0.5 °C)
1	Apathy; lessened mobility; loss of appetite
2	Weight loss; warm extremities; treatable pain without STS
3	Redness of joints (with STS); normal flexibility of extremities
4	Severe STS of joints (plus redness); joint stiffness
5	Untreatable pain; immobility of joints; weight loss > 25%

### Phenotype scoring of CIA in *M. fascicularis*

**Fig. 3:**
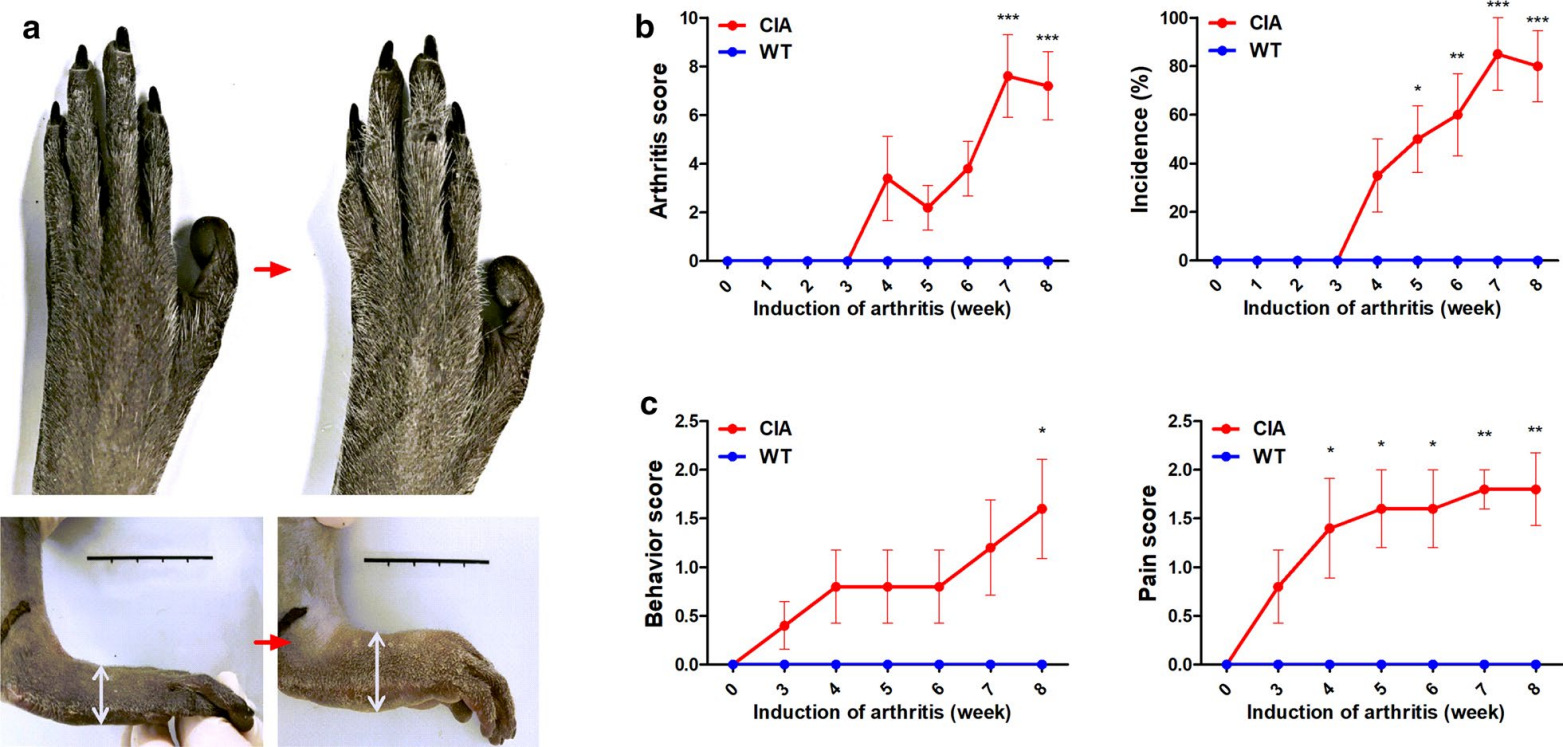
Phenotype scoring of CIA in *M. fascicularis*. **a** Significant changes in CIA were observed compared to the WT. **b** The arthritis score, incidence, and weight are listed in Table 2 (*p < 0.05, **p < 0.01, and ***p < 0.001). **c** The pain and behavior scoring of hyperalgesia were analyzed, as seen in Tables 3 and 4 (*p < 0.05 and **p < 0.01)

Phenotype scoring of the PIP joints and wrists was introduced to fully quantify the assessment of arthritis. The phenotype scoring system included the PIP joints of all limbs except the thumbs (Table 2). In addition, pain and behavior scoring was analyzed from video data captured at 1-min intervals (Tables 3 and 4). Severe edema was observed in the CIA group (Fig. 3a). Also, the pain and behavior scores were higher in the CIA group (Fig. 3c). Phenotype scores of 7–8 were recorded at 8 weeks after CIA induction. There was also a markedly higher incidence of arthritis in the CIA groups (Fig. 3b).

**Table 2:** Phenotype scoring of PIP joint swelling

Arthritis score	Degree of swelling
0	No evidence of swelling
1	Over 0.5 cm swelling of proximal interphalangeal joint compared with 0 week (< 0.5 cm^2)^
2	Score 1 evidence in two proximal interphalangeal joint
3	Score 1 evidence in three proximal interphalangeal joint
4	Score 1 evidence in four proximal interphalangeal joint

**Table 3:** Phenotype scoring of behavior

Ataxia score	Description
0	No evidence of behavior
1	Drag or limp in hind leg
2	Slipped over in cage
3	One’s leg are not working

**Table 4:** Phenotype scoring of pain

Pain score	Description
0	No evidence of pain
1	Tremor of paws
2	Tremor of paws and losing one’s grip
3	Severe tremor of paws and a crouching position

**Fig. 4:**
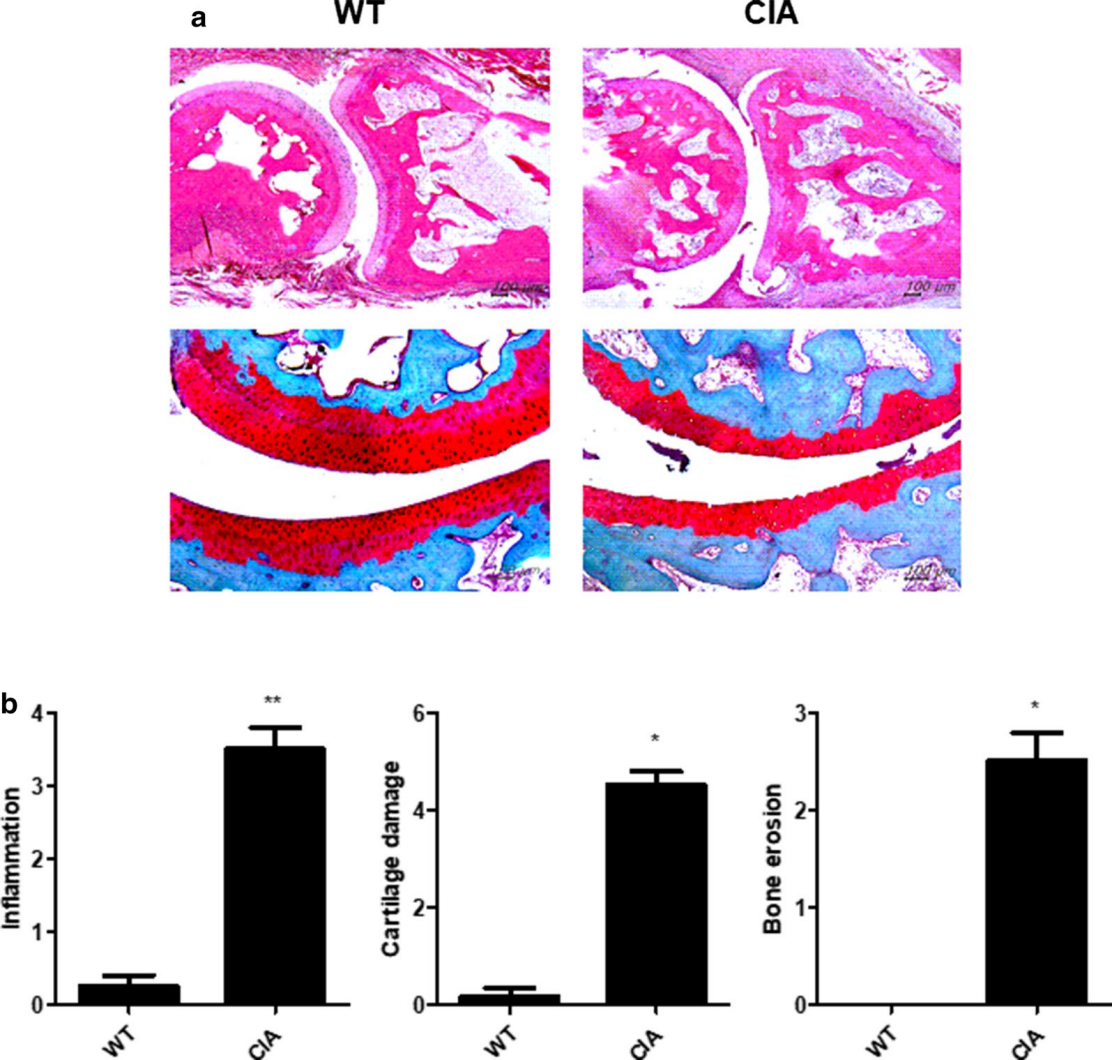
Histopathological analysis of CIA in *M. fascicularis*. **a** Proximal interphalangeal joint tissue samples were acquired from all groups at 8 weeks and stained with H&E and safranin O. **b** Inflammation, cartilage damage, and bone erosion scoring for proximal interphalangeal joint tissue (*p < 0.05 and **p < 0.01)

**Fig. 5:**
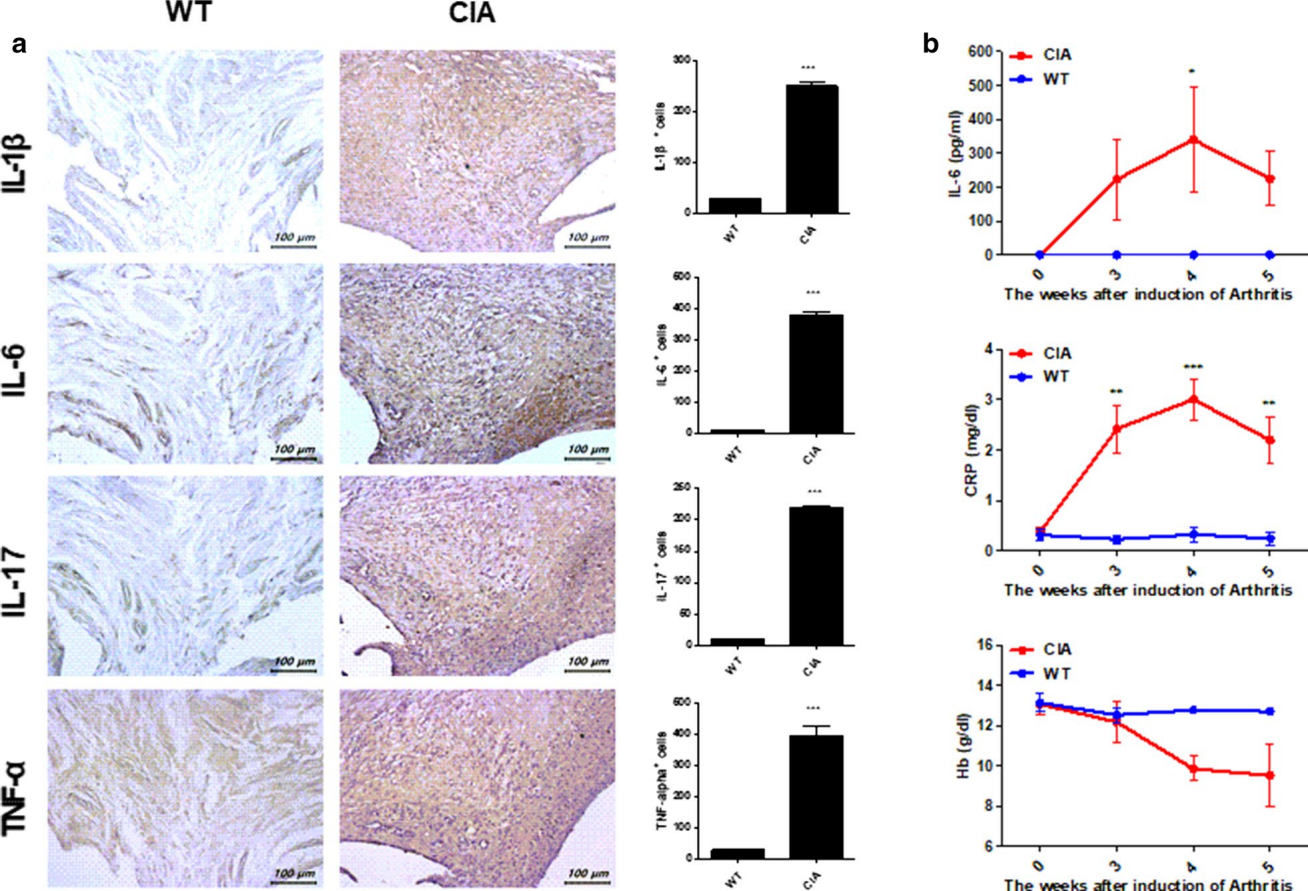
Expression of inflammatory cytokines in synovium and serum. **a** The expression of IL-1β, IL-6, IL-17, and TNF-α in synovium was assessed by immunohistochemistry. Positive cells for each antibody are shown on the right. **b** After 8 weeks, changes in the serum concentration levels were analyzed for IL-6, CRP, and Hb by ELISA, hematology, and serum biochemistry analyses (*p < 0.05, **p < 0.01, and ***p < 0.001)

Safranin O and H&E staining showed increased infiltration of immune cells in the synovium and damage to the cartilage (Fig. 4a). Additionally, the CIA group had a higher score for inflammation, cartilage damage, and bone erosion than the WT group (Fig. 4b). Immunohistochemical staining against pro-inflammatory cytokine- (IL-1β, IL-6, IL-17, and TNF-α) presenting cells were significantly increased in the CIA group compared to the WT group (Fig. 5a). Serum levels of IL-6 and CRP were also significantly higher, and the hemoglobin level was lower in the CIA group (Fig. 5b).

### Inflammatory T cell expansion in PBMCs from *M. fascicularis* with arthritis

**Fig. 6:**
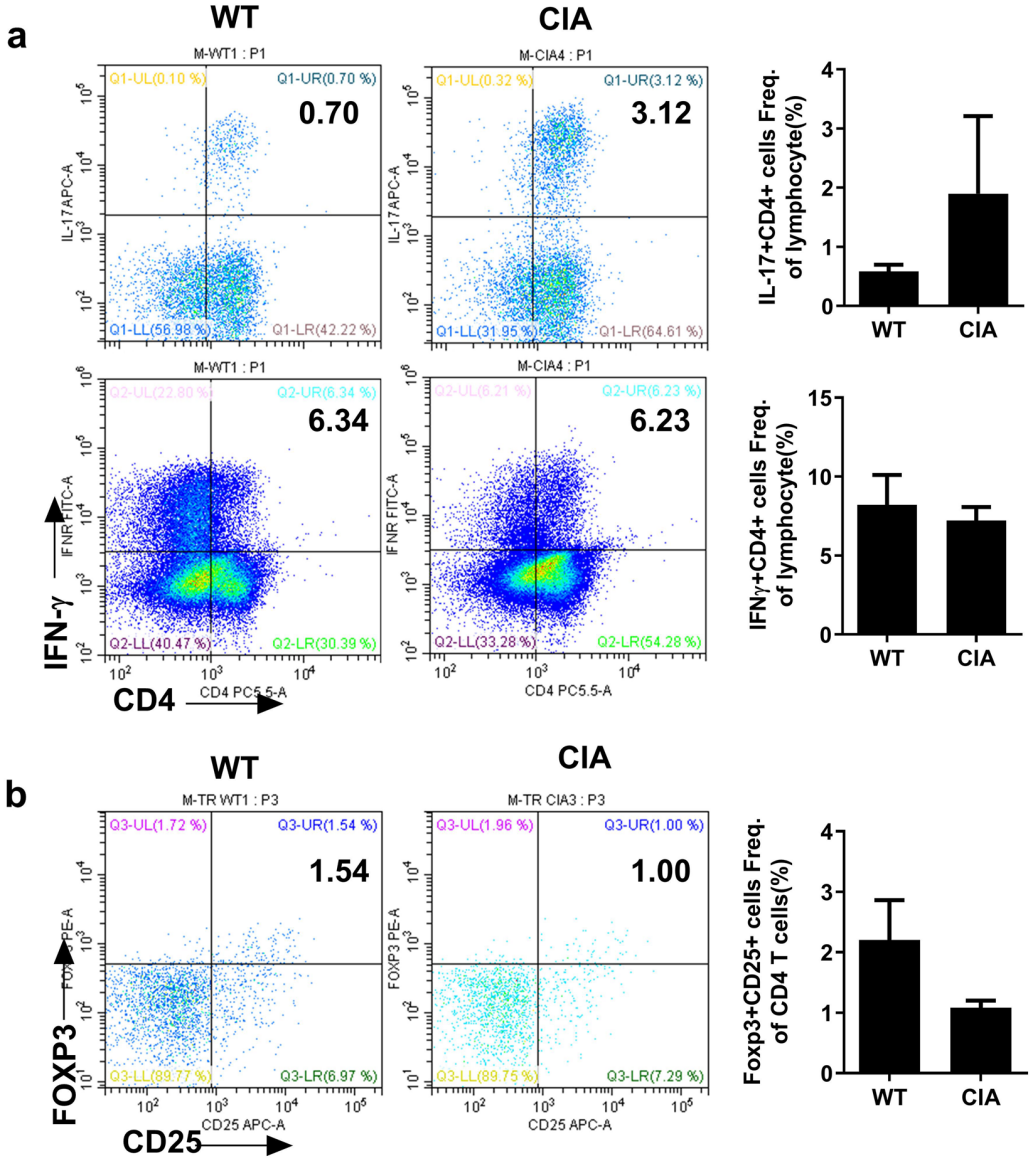
Th17 cell expansion in PBMCs from *M. fascicularis* with arthritis. Peripheral blood mononuclear cells (PBMCs) were isolated at 8 weeks after the induction of arthritis. Expressing cells were analyzed by FACS. **a** T helper cells were stained with PerCP-conjugated anti-CD4 antibody, FITC-conjugated anti-IFNγ antibody, and eFluor 660-conjugated anti-IL-17A antibody (**p < 0.01). **b** Regulatory T cells were stained with PerCP-conjugated anti-CD4 antibody, APC-conjugated anti-CD25 antibody, and PE-conjugated anti-Foxp3 antibody (*p < 0.05)

Next, we analyzed helper T cells and regulatory T cells 6 weeks after induction of arthritis using PBMCs extracted from *M. fascicularis*. The isolated PBMCs were stained with T cell- and cytokine-specific antibodies. The results showed that inflammatory T cells such as IL-17 + CD4 T cells (Th17) was increased in arthritis-induced *M. fascicularis*. On the other hand, IFNγ + CD4 T cells (Th1) decreased (Fig. 6). Both Th1 and Th17 cells contribute to RA pathogenesis, but the role of Th1 cells is controversial [13–15]. Foxp3 + Treg cells decreased slightly in CIA, and Treg cells are known to show correlation with Th17 cells in terms of counter-regulation of Th17 cells [15]. These results suggest that our *M. fascicularis* CIA model is useful for pre-clinical studies of RA.

### Micro-CT analysis of CIA in *M. fascicularis*

**Fig. 7:**
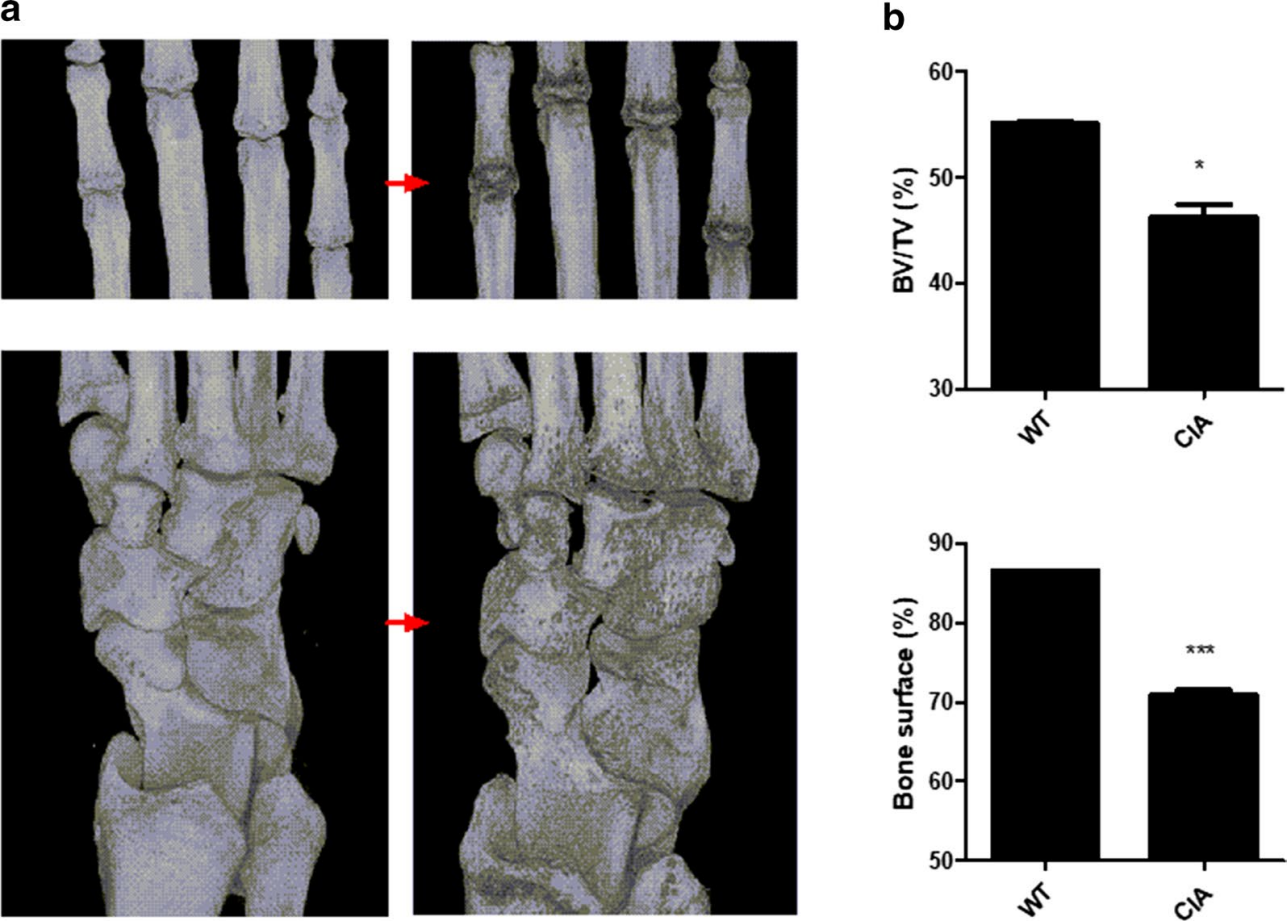
Micro-CT analysis of CIA in *M. fascicularis*. **a** Total samples were scanned using a micro-CT (mCT 35; SCANCO Medical, Wangen-Brüttisellen, Switzerland). **b** Bone surface and bone volume were analyzed using NRecon software (*p < 0.05 and ***p < 0.001). The English in this document has been checked by at least two professional editors, both native speakers of English. For a certificate, please see

Quantitative micro-CT analysis showed the progression of bony damage of the PIP joints and wrist joint in CIA monkeys (Fig. 7a). Quantitative analysis of micro-CT demonstrated that the bone surface and the bone volume/tissue volume ratio were significantly decreased in the CIA group compared to the WT group (Fig. 7b).

## Discussion

In this study, we demonstrated a CIA model in *M. fascicularis* and evaluated this animal RA model according to various clinical, serologic, and histologic parameters. In particular, the combined osteoporotic changes were noted and evaluated in the CIA model in *M. fascicularis*. Although there exist several RA animal models based on primates, these RA animal models were not standardized to evaluate the degree of arthritis.

*Macaca mulatta*-based RA animal models were the first primate models used. However, arthritis induction was successful in only 70% of *M. mulatta*, and arthritis-resistant rhesus monkeys possess a specific MHC allele, *Mamu*-B*01 [4, 11], which is identical to *Gogo*-B*01 in *M. fascicularis*. The present study showed that *Gogo*-B*01 allele-positive *M. fascicularis* were susceptible to CIA induction. This suggests that the arthritis-resistant MHC allele may differ between species, even when they belong to the same genus. Previous studies have demonstrated CIA induction in *M. fascicularis* [6–9], however, there has been no evaluation of the genetic background of the arthritis-resistant MHC allele. The present study showed that the specific MHC alleles known to play crucial roles in the induction of CIA in *M. mulatta* were not active in *M. fascicularis*. This is the first report to demonstrate the influence of the genetic background in arthritis induction in *M. fascicularis*.

Previous studies have demonstrated CIA induction in *M. fascicularis*, and have shown the therapeutic effects of IL-6 neutralizing agents in CIA [6, 7, 16–18]. However, these studies did not use a standardized scoring system or conduct behavior and pain analysis, and did not evaluate bone mineral density. In the present study, we focused on evaluating clinical, phenotypical, behavior, and pain parameters in a *M. fascicularis*-based CIA model. Osteoporosis, a well-known co-morbidity of RA, was also quantitatively assessed by micro-CT scanning, which is used in osteoporosis evaluation [19]. This information could expand the use of *M. fascicularis*-based RA animal models to evaluate and demonstrate the therapeutic effects of novel agents in aspects of RA-related osteoporosis.

The present study has several limitations. First, although we set the clinical/phenotypical/behavior/pain score in a CIA model using *M. fascicularis*, further studies are needed to clarify the usefulness of the scoring system. Several pain and behavior assessment tools are available for rodent or mice models such as the Von Frey or Randall Selitto test [20]; these methods can be modified for use in monkey models in further studies. Second, although the positive *Gogo*-B*01 allele in *M. fascicularis* did not show resistance to CIA induction, the induction rate for arthritis was approximately 80%. This suggests another MHC allele may be responsible for arthritis resistance in *M. fascicularis*, and the specific arthritis-resistant MHC allele was not demonstrated in the present study.

## Conclusion

The present study aimed to establish a novel scoring system for a *M. fascicularis*-based CIA and RA animal model that can be used to score clinical data, but also to score for behavior and pain. In addition, osteoporotic changes were clearly observed in this model, and therefore, a *M. fascicularis*-based CIA model could assess the effects of novel RA medication on RA-mediated osteoporosis.

## Data Availability

All data are available within the manuscript or upon request to the authors.

## Abbreviations

CIA: Collagen-induced arthritis
MHC: Major histocompatibility complex
RA: Rheumatoid arthritis
